# Targeting mucosal immunity in malaria control: the underexplored role of IgA

**DOI:** 10.3389/fmala.2025.1557371

**Published:** 2025-06-24

**Authors:** Haruna Muwonge, Isaac Ssewanyana, Adoke Yeka, Pauline Byakika-Kibwika

**Affiliations:** 1 Department of Physiology, College of Health Sciences, Makerere University, Kampala, Uganda; 2 Inter-Epidemic Consortium for Epidemic Research, Makerere University Lung Institute (MLI), Kampala, Uganda; 3 Department of Laboratory Services, National Health Laboratory and Diagnostic Services (NHLDS), formerly Central Public Health Laboratories (CPHL), Kampala, Uganda; 4 Mbarara University of Science and Technology (MUST), Mbarara, Uganda

**Keywords:** immunoglobulinA (IgA), mucosal immunity, malaria vaccines, *Plasmodium falciparum*, sporozoite neutralization, complement activation, mucosal vaccine delivery, malaria transmission blocking

## Abstract

Malaria remains a global health crisis, causing an estimated 263 million cases and 597,000 deaths in 2023. Current measures—including insecticide-treated nets, ACTs, and the RTS,S vaccine—have stalled in reducing mortality, highlighting the need for novel strategies. While studies IgG and IgM have dominated malaria immunology research, recent data reveal a broader role for Immunoglobulin A (IgA). Evidence suggests that IgA can block parasite entry, activate complement, and modulate inflammation, although its protective efficacy has yet to be established. This review synthesizes the emerging literature on sporozoite- and merozoite-specific IgA responses, examines how IgA arises in a “non-mucosal” infection like malaria, and explores vaccine platforms-oral, nasal, or prime-boost-that might harness IgA alongside IgG. We also identify critical gaps in correlating IgA levels with clinical immunity, emphasizing the need for specialized animal models and longitudinal human cohorts. Ultimately, leveraging IgA-driven mucosal immunity could significantly reinforce existing malaria interventions by preventing parasite establishment at mucosal or skin interfaces. By uniting mucosal and systemic immunity, research on IgA-based vaccines promises a next-generation approach to reducing malaria transmission, thereby creating a path towards global eradication.

## 1 Introduction

Malaria remains one of the world’s most serious infectious diseases, with an estimated 263 million cases and 597,000 deaths in 2023 alone. The World Health Organization (WHO) African Region continues to bear the greatest burden, accounting for 94% of malaria cases and 95% of malaria deaths globally (World Health Organization, 2024). Although existing control measures such as, insecticide-treated nets (ITNs), artemisinin-based combination therapies (ACTs), and the gradual deployment of the RTS,S malaria vaccine have reduced morbidity in some regions (Gething et al., 2016; Weiss et al., 2019), progress in lowering malaria mortality has stalled in recent years. This plateau underscores the fragility of current gains and the pressing need for additional strategies to address mounting insecticide resistance (Lindblade et al., 2015; N’Guessan et al., 2007) and emerging antimalarial drug resistance, which are further complicated by socioeconomic constraints and shifting transmission dynamics (Qiao et al., 2021). In this context, WHO has emphasized the importance of adaptive approaches (Kayentao et al., 2018), including innovative vaccine designs that transcend the partial, short-lived protection elicited by current IgG-centric platforms such as RTS,S (Suau et al., 2021).

Historically, most malaria immunology research has focused on Immunoglobulin G (IgG) and IgM, given their established roles in neutralizing blood-stage (merozoite) parasites and targeting sporozoites in the skin or liver (Hopp et al., 2021; Rosenkranz et al., 2023). By comparison, Immunoglobulin A (IgA), primarily associated with mucosal surfaces like the gut and respiratory tract was once considered peripheral to malaria (Berry et al., 2021; Macpherson et al., 2008). However, shifting perspectives reveal that parasite-specific IgA can indeed arise in natural and experimental malaria infections (Berry et al., 2021; Tan et al., 2021), prompting new questions about its potential to neutralize sporozoites early in infection, engage the alternative and lectin complement pathways, and modulate inflammatory responses (Rogier et al., 2022; Shi et al., 2011). Tan et al. demonstrated that functional human IgA targets a conserved site on Plasmodium falciparum sporozoites (Tan et al., 2021), offering compelling evidence that IgA can contribute to neutralizing infection at the earliest stages. Yet, robust data on how consistently IgA confers clinical protection remain scarce, exposing a key research gap that next-generation vaccine efforts may need to address.

Building on these observations, there is growing recognition that mucosal immunity -particularly the IgA response- could serve as a vital adjunct to systemic IgG-based interventions, blocking parasites at entry points and potentially enhancing overall immune clearance. The idea of leveraging IgA in oral or nasal formulations is gaining traction, as these delivery routes could induce local immune responses that obstruct *Plasmodium falciparum* before it progresses to the blood stage. Such an approach not only complements IgG-mediated strategies (e.g., RTS,S) but also bolsters integrated malaria control programs, which combine ITNs, ACTs, and other public health measures to halt transmission.

In the sections that follow, we first examine the foundational biology of mucosal compartments and secretory IgA, shedding light on how this antibody can function both locally and systemically. We then evaluate emerging data on IgA-mediated protection against malaria, including evidence from controlled human malaria infection (CHMI) studies, and highlight the key data gaps that must be addressed through further research. Finally, we discuss how an integrated approach -one that leverages synergy between mucosal IgA and systemic IgG responses- may overcome the current stagnation in malaria mortality reductions and reinvigorate global eradication efforts.

## 2 IgA in the immune system and malaria immunity

### 2.1 Overview of IgA and mucosal immunity

Immunoglobulin A (IgA) is classically recognized for its key role at mucosal surfaces in the gastrointestinal, respiratory, and urogenital tracts (Boyaka, 2017). These mucosal surfaces represent the largest interface between the body and the external environment, covering over 400 m^2^ of surface area (Lamichhane et al., 2014). Given that more than 80% of infectious pathogens initiate infection at these sites (Tang and Chen, 2010), the mucosal immune system is uniquely adapted to provide first-line protection against invading microbes.

**FIGURE 1:**
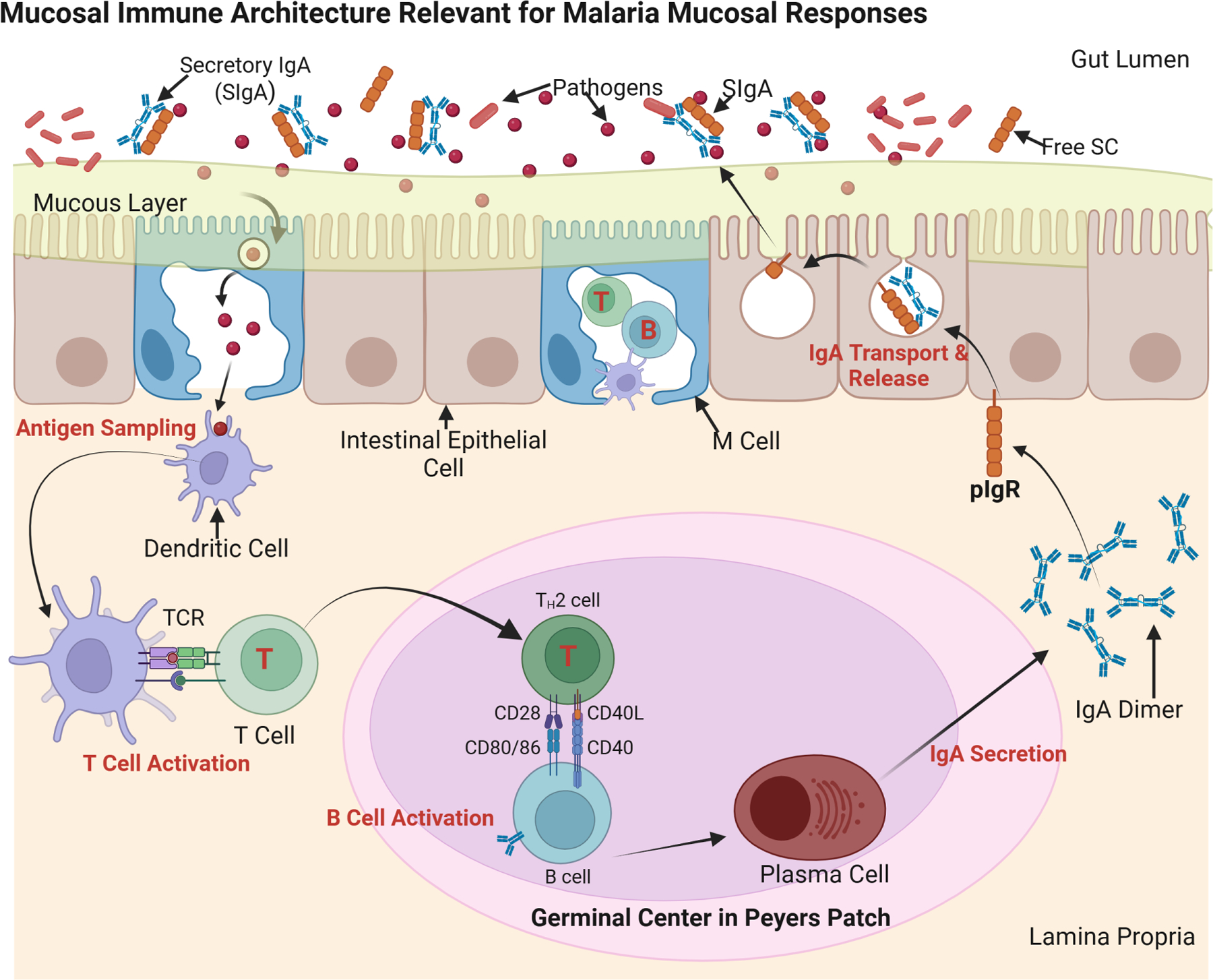
Mucosal immune architecture and IgA production. This schematic illustrates secretory IgA (SIgA) induction in gut-associated lymphoid tissue (GALT), a model for potential malaria mucosal immunity. M (microfold) cells transport antigens from the gut lumen to dendritic cells (DCs), which activate T helper (TH) cells and drive B cell class switching to IgA in Peyer’s patches. Plasma cells secrete polymeric IgA (pIgA), which is transported across epithelial cells via the polymeric immunoglobulin receptor (pIgR) and released as SIgA. SIgA neutralizes pathogens at mucosal surfaces, maintaining homeostasis and preventing infection. Source: Created with BioRender.com.

Multiple layers of defense operate at mucosal surfaces to prevent pathogen entry. Tight junctions between epithelial cells form physical barriers, mucus traps and immobilizes microbes, and a diverse array of antimicrobial peptides further impedes infection. Within this environment, secretory IgA (SIgA) serves as a frontline immunoglobulin, capable of neutralizing and agglutinating pathogens before they penetrate the epithelium (Corthésy, 2013). SIgA contains a secretory component that protects it from enzymatic degradation, allowing it to function effectively in hostile environments such as the gut lumen (Figure 1).

Specialized mucosa-associated lymphoid tissue (MALT) coordinates local immune responses at mucosal sites. In the gut-associated lymphoid tissue (GALT), Peyer’s patches house M (microfold) cells, which transport luminal antigens to underlying dendritic cells and B lymphocytes (Fujihashi et al., 2001). This antigen presentation drives B cell class switching to IgA and the differentiation of IgA-secreting plasma cells. Analogous immune structures are found in the respiratory tract, including bronchus-associated lymphoid tissue (BALT) and nasal-associated lymphoid tissue (NALT), which regulate mucosal immunity in the lungs and upper airways (Bienenstock and McDermott, 2005). These tissues play a dual role: generating immune tolerance to non-harmful antigens while mounting rapid, localized responses against pathogens to limit their dissemination.

Although IgA is primarily recognized for its mucosal functions, it also circulates as a monomer in the bloodstream, where it ranks second in serum levels after IgG (Macpherson et al., 2008). The systemic functions of IgA remain less well characterized than its mucosal roles, but emerging evidence suggests that both mucosal and circulating IgA contribute to immune defense in various infectious diseases. Understanding the mechanisms by which IgA functions beyond mucosal surfaces could provide new insights into its potential role in malaria immunity and vaccine design.

### 2.2 Malaria-specific IgA responses: pre-erythrocytic stage and merozoite

Despite Plasmodium falciparum mainly infecting the liver (pre-erythrocytic) and red blood cells (merozoite) -tissues not typically defined as mucosal- recent studies highlight a potentially critical role for IgA in malaria. Controlled human malaria infection (CHMI) trials have demonstrated robust sporozoite-specific IgA responses, particularly against the circumsporozoite protein (CSP) (Berry et al., 2021). *In vitro* and mouse models further show that CSP-specific IgA monoclonal antibodies can reduce hepatocyte invasion, thus lowering liver parasite burden (Shi et al., 2011; Tan et al., 2021). Blocking parasites at this early pre-erythrocytic stage may be crucial for preventing subsequent merozoite proliferation. In the RTS,S phase 3 trial, Suau et al. (2021) found that CSP-specific IgG responses were 3–5-fold higher than IgA levels in vaccinated African children (Suau et al., 2021). However, individuals with detectable IgA exhibited unique transcriptional profiles suggesting additional mucosal priming. These data imply that while IgA levels may be lower quantitatively, they could provide qualitative advantages under certain exposure conditions.

Interestingly, merozoite-specific IgA has also been detected, although its protective efficacy remains inconsistent. Some investigations link IgA targeting merozoite surface proteins (MSPs) to reduced erythrocyte invasion (Shi et al., 2011), while others suggest it mainly modulates inflammation rather than acting to decrease parasite replication (Berry et al., 2021). Recent evidence also shows that IgA specific to erythrocyte-binding ligand region VI (rVI) in P. falciparum confers tissue protection in murine models without significantly reducing parasitemia (Deore et al., 2019). Immunization with synthetic rVI peptides (Mpep3/Mpep4) led to elevated IgA levels, which were associated with improved lung and cerebral pathology in Plasmodium berghei-infected mice. In human sera from endemic regions, approximately 32% showed IgA reactivity to these rVI epitopes, reinforcing their relevance in natural infection.Pinpointing whether merozoite-targeted IgA confers substantive protection or primarily influences disease pathology warrants further human clinical studies, as most data currently derive from animal or *in vitro* systems.

### 2.3 Origins of IgA in “non-mucosal” malaria

A pressing question is how a “mucosal-type” antibody arises in an infection transmitted by mosquito bite and progressing through the liver and bloodstream. One hypothesis proposes that the act of sporozoite deposition in the skin elicits local dendritic cell activity or unique cytokine signals (e.g., TGF-β, IL-10), fostering IgA class switching (Berry et al., 2021; Boyaka, 2017; Macpherson et al., 2008). Recent transcriptomic studies from controlled human malaria infection (CHMI) trials suggest upregulation of AICDA and IL-10 in peripheral B cells during early liver-stage infection, implying potential systemic class switching to IgA even outside mucosal inductive sites. Moreover, skin-resident dendritic cells have been shown to migrate to draining lymph nodes and drive IgA responses via TGF-β and BAFF signaling (Berry et al., 2021; Macpherson et al., 2008). These findings support the plausibility of systemic IgA induction, albeit through unconventional routes. Other theories involve crosstalk between gut- or nasopharynx-associated lymphoid tissue and systemic immune compartments, which might amplify IgA-producing B cells during liver-stage or early merozoite infection. However, robust clinical data establishing these pathways remain limited, underscoring the need for deeper immunological investigations.

Further evidence for IgA induction in malaria comes from a phase 3 trial of RTS,S/AS01E, where sporozoite-specific IgA and even IgA against non-vaccine antigens emerged in naturally exposed African children (Suau et al., 2021). Although no significant correlation with protection was observed, these findings confirm that IgA responses can arise *in vivo*. Whether such IgA meaningfully augments vaccine efficacy or complements naturally acquired immunity in endemic areas is an open question.

### 2.4 Significance for vaccine development

Given the indication that sporozoite-targeted IgA can inhibit early infection, interest has grown in mucosal or prime–boost vaccine platforms capable of inducing potent IgA alongside established IgG responses (Suau et al., 2021). Current RTS,S formulations already elicit some IgA (Suau et al., 2021), but determining how to maximize this response - and whether it translates into clinical protection - remains a priority. Additional synergy with IgG mechanisms, such as antibody-dependent cellular cytotoxicity or complement activation, could potentially enhance overall efficacy (Boyaka, 2017; Shi et al., 2011). Moreover, the capacity of IgA to bind parasite antigens beyond CSP hints that vaccination might be tailored to amplify naturally acquired immunity in endemic settings. One example includes GMZ2.6c, a chimeric vaccine candidate incorporating GLURP, MSP-3, and Pfs48/45 antigens, which elicits naturally acquired antibody responses, including IgA in individuals from malaria-endemic areas of Brazil (Baptista et al., 2022). These data therefore support the idea that multi-component vaccines could broaden the immune repertoire beyond canonical targets like CSP and potentially enhance mucosal as well as systemic responses.

Ultimately, merozoite-specific IgA could also play a role in merozoite control, though data on its protective potency is scanty. Notably, most licensed malaria vaccines rely heavily on IgG-driven mechanisms for merozoite clearance. It remains uncertain whether IgA offers additive or redundant protection when strong IgG responses are present. As such, a dual strategy combining IgG-mediated merozoite clearance with IgA-driven pre-erythrocytic blockade may be advantageous in scenarios where IgG responses are suboptimal or where early mucosal interception is critical.

## 3 IgA effector mechanisms in malaria

### 3.1 Neutralization of parasites

**FIGURE 2:**
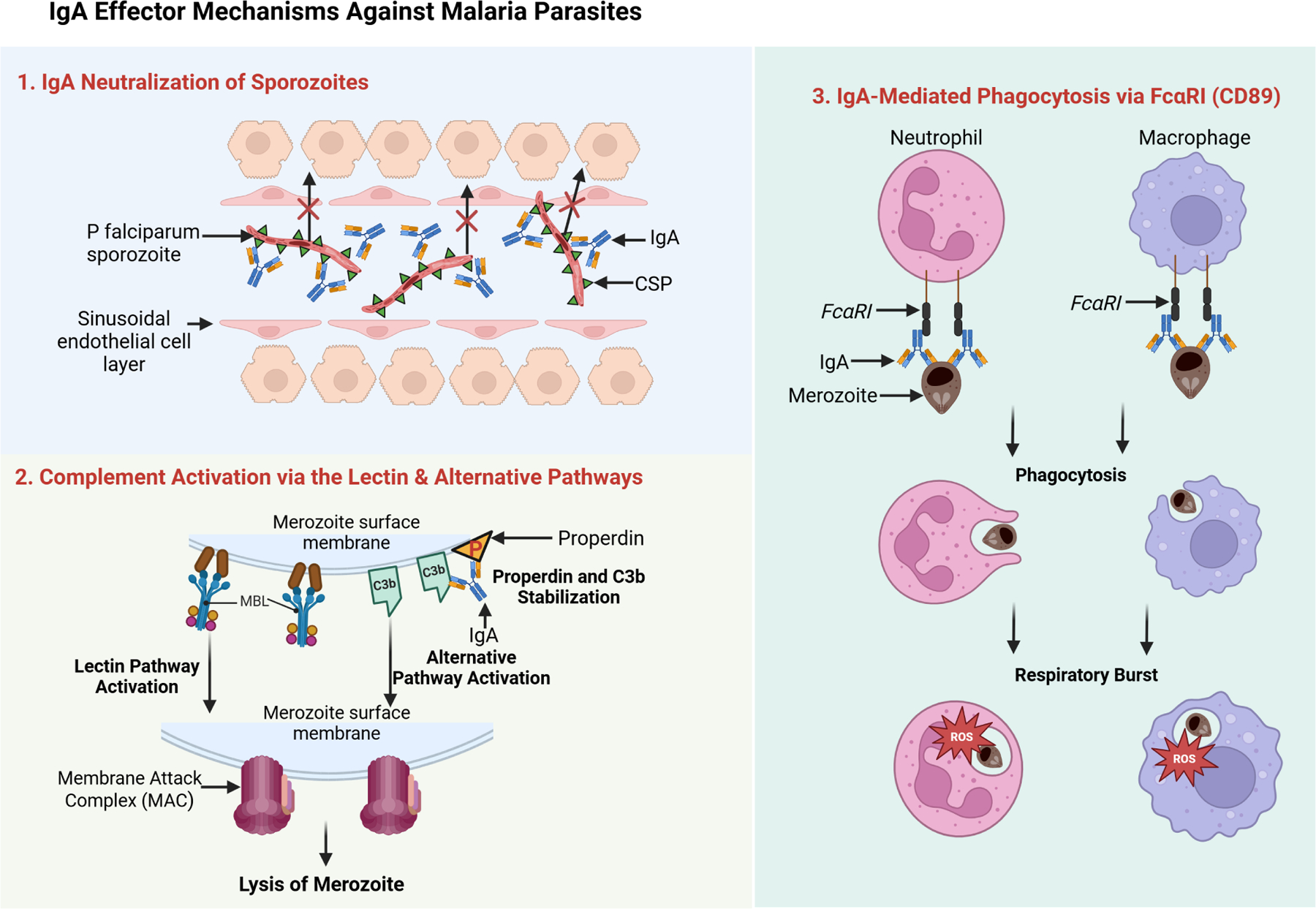
IgA effector mechanisms against malaria parasites. This schematic illustrates three key IgA-mediated defense strategies. 1. First, IgA neutralizes *Plasmodium* sporozoites by binding surface antigens such as the circumsporozoite protein (CSP), thereby blocking liver-cell invasion. 2. Second, IgA triggers complement activation via the lectin and alternative pathways: IgA-coated parasites recruit mannose-binding lectin (MBL), leading to opsonization and membrane attack complex (MAC) formation, while properdin and C3b stabilize the alternative pathway. 3. Third, IgA’s Fc region binds FcaRI (CD89) on neutrophils and macrophages, promoting phagocytosis and respiratory burst. Source: Created with BioRender.com.

IgA commonly protects mucosal surfaces by directly binding pathogens and preventing their adherence or invasion (Boyaka, 2017) as demonstrated in Figure 2. Although Plasmodium falciparum typically infects the liver and blood rather than traditional mucosal sites, *in vitro* and animal studies indicate that IgA can still coat sporozoites or merozoites, blocking their entry into host cells (Tan et al., 2021). For instance, a circumsporozoite protein (CSP)–specific IgA monoclonal inhibited liver-stage invasion in a mouse model, suggesting that neutralization in the early (pre-erythrocytic) phase could be pivotal for preventing subsequent merozoite infection (Tan et al., 2021). Importantly, Tan et al. identified a conserved site on the sporozoite that can be targeted by IgA, reinforcing the feasibility of developing broadly protective antibodies for vaccine or therapeutic purposes. This mechanism typically elicits minimal inflammation, as IgA-bound parasites are passively prevented from further replication—a crucial advantage for achieving sterile protection.

### 3.2 Activation of complement pathways

When parasites evade initial IgA binding, complement activation serves as a secondary defense. Unlike IgG and IgM - which effectively initiate the classical pathway - IgA generally has low affinity for C1q and thus weakly triggers the classical route (Maillard et al., 2015). However, IgA-coated targets can drive the alternative pathway by stabilizing C3b and properdin deposition (Maillard et al., 2015) as demonstrated in Figure 2. Additionally, research in IgA nephropathy suggests that certain IgA-containing immune complexes may engage the lectin pathway; for instance, through interactions with Mannose-Binding Lectin (MBL) (Endo et al., 1998; Hisano et al., 2001). Although this lectin-mediated route in malaria is less clearly established and may depend on specific glycosylation patterns of both IgA and parasite antigens (Ding et al., 2022), complement activation of any type can culminate in opsonization and formation of membrane attack complexes (Kurtovic et al., 2018; Owuor et al., 2008). Epidemiological data linking MBL, factor H, and properdin to reduced malaria severity (Rathnayake et al., 2021) underscore the potential significance of these pathways for *P. falciparum* clearance.

### 3.3 Fc Receptors and phagocytosis

Beyond complement, IgA-coated parasites can be internalized by immune cells expressing FcαRI (CD89) (Figure 2), such as neutrophils, monocytes, and macrophages (Pleass, 2009). This “opsonization” promotes phagocytosis, destruction within phagolysosomes, and release of reactive oxygen species or inflammatory mediators (Nziza et al., 2023). While IgG-mediated opsonization in malaria is well documented and correlates with vaccine-induced protection (Nziza et al., 2023), *in vivo* evidence for IgA’s role remains limited, partly because standard murine models lack human FcαRI (Tan et al., 2021). Nonetheless, in human populations, harnessing IgA-FcαRI interactions could be particularly valuable for targeting *P. falciparum* during both pre-erythrocytic and blood stages.

## 4 Integrating mucosal immunity into malaria eradication strategies

### 4.1 Rationale for a mucosal approach

Building on the evidence that IgA can neutralize Plasmodium falciparum sporozoites and potentially reduce merozoite infection, it is logical to explore how mucosal vaccination might amplify these protective responses. This section outlines the rationale for a mucosal approach and describes specific strategies for delivering malaria antigens to mucosal surfaces, where IgA-mediated defenses are most potent.

**FIGURE 3:**
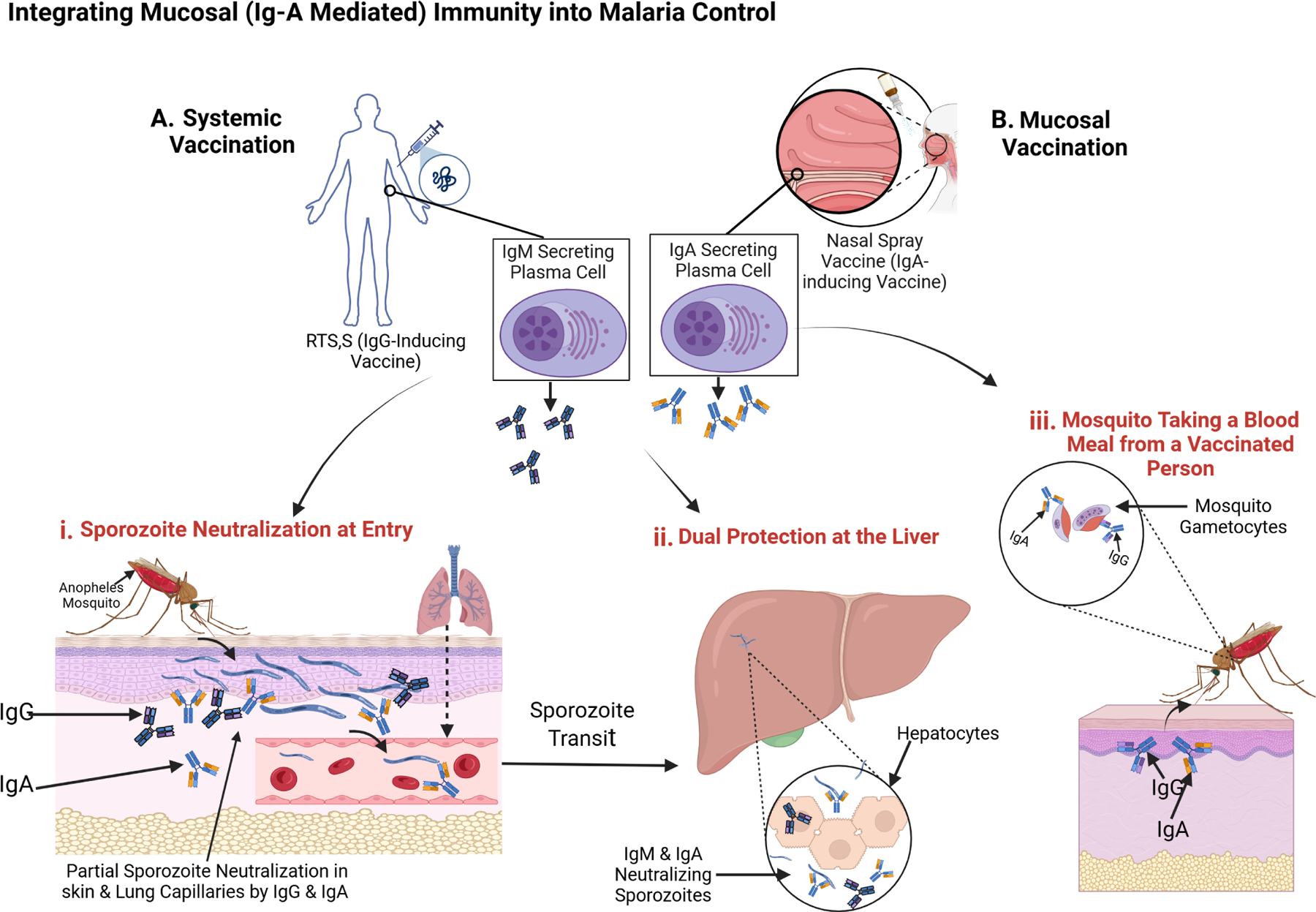
Integrating Mucosal (IgA-Mediated) Immunity into Malaria Control. This diagram illustrates how systemic IgG (dark blue Y-shapes) and mucosal IgA (light blue Y-shapes) can work in tandem to thwart *Plasmodium falciparum* at multiple stages of the parasite life cycle. **(A)** Systemic Vaccination with RTS,S induces IgG-producing plasma cells, while **(b)** Mucosal Vaccination via intranasal spray induces IgA-secreting plasma cells in respiratory-associated lymphoid tissue. These immune responses contribute to: (i) Sporozoite Neutralization at Entry – IgG and IgA bind and partially neutralize sporozoites in the skin and lung capillaries shortly after a mosquito bite, limiting their dissemination. (ii) Dual Protection at the Liver – Sporozoites that escape initial neutralization are further targeted by IgG, IgM, and IgA during liver-stage transit, preventing hepatocyte invasion. (iii) Transmission Blocking – When a mosquito feeds on a vaccinated individual, it ingests gametocytes coated with IgG and IgA, interfering with parasite development in the mosquito midgut and reducing transmission. Source: Created with BioRender.com.

Eradicating malaria requires multifaceted interventions that target both human and mosquito stages of the parasite. Although insecticide-treated nets, indoor residual spraying, and artemisinin-based combination therapies have lowered incidence in some regions, P. falciparum persists in a significant parasite reservoir (WHO, 2024). Moreover, the RTS,S/AS01 vaccine offers only partial efficacy (30–50% in the first year) against the circumsporozoite protein (CSP), and its protection diminishes rapidly (Suau et al., 2021). Mucosal vaccination presents an opportunity to augment or complement these measures by eliciting IgA - a key immunoglobulin for neutralizing pathogens at their entry points (Boyaka, 2017) as shown in Figure 3. Given that sporozoites traverse skin and lung microvasculature before reaching the liver, boosting IgA in these tissues could intercept parasites early and reduce the overall parasite burden in the community.

### 4.2 Potential strategies for mucosal vaccine delivery

#### 4.2.1 Oral or intranasal vaccination

Oral and intranasal platforms have proven effective in other infectious diseases such as, polio and influenza, by provoking localized IgA responses in the gut- or nasopharynx-associated lymphoid tissues (Boyaka, 2017). For malaria, an intranasal vaccine could theoretically intercept sporozoites that lodge in the lung microvasculature (Figure 3), whereas an oral vaccine might stimulate gut-associated lymphoid tissue, seeding IgA-producing B cells throughout mucosal and systemic sites (Macpherson et al., 2008). By establishing a sporozoite-specific IgA response both locally and in circulation, these approaches could limit parasite migration to the liver and thus diminish the reservoir available for mosquito transmission.

#### 4.2.2 Epicutaneous (skin) immunization

Skin-based immunization targets the extensive network of immune cells, including Langerhans cells, that reside in the epidermis and dermis (Arakawa et al., 2009). Delivering malaria antigens through microneedle patches or topical applications could generate local IgA at or near the mosquito bite site, directly neutralizing sporozoites before they disseminate (Figure 3). Although skin vaccination has shown promise in preclinical trials for certain pathogens, further research is needed to confirm its efficacy and feasibility against *P. falciparum*, especially in regions with limited healthcare infrastructure.

#### 4.2.3 Combination prime–boost approaches

A heterologous prime–boost regimen can merge the advantages of systemic and mucosal immunization. An initial intramuscular injection - for example, with RTS,S/AS01 - induces a robust IgG and T-cell response, which a subsequent oral or intranasal boost can supplement by promoting IgA-secreting plasma cells in mucosal tissues (Boyaka, 2017). This layered approach potentially offers broader immunological coverage, blocking parasites at multiple junctures of infection and transmission while also preserving the convenience and familiarity of intramuscular vaccination.

#### 4.2.4 Targeting transmission stages

Mucosal immunization may also enhance efforts to block malaria transmission by eliciting IgA directed against parasite sexual forms in the mosquito midgut (Arakawa et al., 2009). Because IgA is relatively resistant to proteolysis (Boyaka, 2017), these antibodies might persist longer than IgG within the mosquito gut, neutralizing gametocytes, gametes, or ookinetes (Figure 3). Consequently, a strong IgA response in the human host could disrupt the parasite’s development cycle in mosquitoes, lowering overall transmission and contributing to population-level control.

## 5 Existing challenges and future directions

### 5.1 Understanding the induction and variability of IgA

A key unresolved issue is how *P. falciparum* infection or vaccination triggers IgA. Malaria is not classically considered a mucosal disease, so the factors leading to IgA class-switching-whether sporozoite deposition in the skin, liver-stage replication, or systemic cytokine cues remain unclear (Berry et al., 2021; Boyaka, 2017). Furthermore, not all naturally exposed individuals mount robust IgA responses; some studies report highly variable sporozoite-specific IgA levels across different ages or epidemiological settings (Berry et al., 2021). Notably, repeated malaria infection and recovery cycles in murine models led to progressively increasing levels of parasite-specific IgA and circulating TGF-β; an immunoregulatory cytokine known to promote IgA class-switching (Deore et al., 2019). These findings simulate endemic conditions and suggest that chronic or repeated exposure may be critical for stable IgA responses. Establishing whether these variable IgA responses correlate with reduced parasitemia or clinical protection will be essential. Longitudinal cohort studies investigating when and why IgA emerges, and how this correlates with parasite burden, could elucidate optimal strategies for artificially inducing it through vaccination.

### 5.2 Advancing mucosal vaccine design

Another significant challenge is eliciting sustained IgA via vaccination. Most licensed vaccines given intramuscularly induce IgG-dominant responses rather than IgA (Boyaka, 2017). Mucosal delivery routes, such as intranasal or oral vaccination, must overcome *multiple biological barriers*, including enzyme-rich environments and the need for safe yet potent adjuvants that stimulate IgA. Ongoing innovations, such as live attenuated vectors, microneedle patches, or specialized prime–boost strategies may improve IgA durability. However, ensuring these platforms remain stable and practical in low-resource environments remains a formidable task.

### 5.3 Preclinical models and translational research

Standard mouse models do not accurately reproduce human IgA biology, largely because they lack the human FcαRI receptor (Tan et al., 2021). Transgenic mice expressing human IgA or FcαRI can partially address this gap, but non-human primates may be needed for closer approximation to human immune responses. Meanwhile, human challenge trials (CHMI) allow a controlled examination of candidate IgA-based vaccines or therapeutic antibodies. Laboratory assays (e.g., mosquito membrane feeding tests, *in vitro* liver-stage neutralization) can also clarify how effectively IgA reduces parasite viability or disrupts transmission.

### 5.4 Novel IgA-based interventions

Monoclonal IgA therapy exemplifies a frontier approach, with certain IgA clones shown to neutralize sporozoites or reduce liver infection (Shi et al., 2011; Tan et al., 2021). Though IgA’s shorter serum half-life poses challenges, protein engineering could enhance stability, half-life, and effector functions. The discovery that functional human IgA can recognize conserved epitopes on the sporozoite surface (Tan et al., 2021) presents a valuable opportunity for designing universal IgA-based therapeutics with broad strain coverage. Delivering IgA as an inhalant, oral capsule, or skin patch- especially around high-transmission seasons-could provide temporary but potent protection at relevant mucosal sites.

### 5.5 Safety, implementation, and public health integration

While IgA is often less inflammatory than IgG, immune complexes containing high levels of IgA can sometimes provoke tissue damage in diseases such as IgA nephropathy. Currently, there is no evidence suggesting malaria-related IgA causes such pathology, but vigilance is essential as researchers explore ways to boost IgA responses (Boyaka, 2017). Furthermore, integrating new IgA-based approaches into existing malaria control programs requires alignment with vaccination schedules, community acceptance, and health-system readiness (Kayentao et al., 2018). Where feasible, needle-free immunization (oral or intranasal) may improve accessibility and uptake, but any new method must demonstrate clear advantages over the status quo to justify changes in policy and practice.

## 6 Conclusion

IgA, once considered less critical than IgG or IgM in malaria immunity, has emerged as a key immunoglobulin that can neutralize Plasmodium falciparum sporozoites early, engage complement pathways, and support Fc-mediated parasite clearance. While its canonical function involves protecting mucosal surfaces, IgA can also circulate systemically and bind antigens beyond those targeted by existing vaccines.

Mucosal vaccination platforms, such as oral, intranasal, or combination prime–boost approaches have the potential to generate targeted IgA responses where parasites first gain entry, and work synergistically with systemic IgG. Leveraging IgA in both early sporozoite interception and sexual-stage parasite neutralization could significantly reduce malaria transmission.

Despite promising data, critical questions remain about IgA’s induction and long-term efficacy in endemic settings, including whether it provides unique or redundant protection in the presence of robust IgG responses. Improved animal models reflecting human IgA biology, and comparative studies quantifying IgA versus IgG efficacy, are urgently needed. Moving forward, clarifying IgA’s mechanisms, optimizing vaccine designs, and validating these strategies in the field will help overcome current limitations in malaria control and propel global eradication efforts.
